# A Broad Influenza Vaccine Based on a Heat-Activated, Tissue-Restricted Replication-Competent Herpesvirus

**DOI:** 10.3390/vaccines12070703

**Published:** 2024-06-23

**Authors:** Nuria Vilaboa, David C. Bloom, William Canty, Richard Voellmy

**Affiliations:** 1 Hospital Universitario La Paz-IdiPAZ, 28046 Madrid, Spain; nuria.vilaboa@salud.madrid.org; 2 CIBER de Bioingenieria, Biomateriales y Nanomedicina, CIBER-BBN, 28046 Madrid, Spain; 3 Department of Molecular Genetics & Microbiology, University of Florida College of Medicine, Gainesville, FL 32610-0266, USA; dbloom@ufl.edu (D.C.B.); williamcanty@ufl.edu (W.C.); 4 HSF Pharmaceuticals SA, 1814 La Tour-de-Peilz, Switzerland

**Keywords:** vaccine, vectored vaccine, influenza, herpesvirus, replicating, heat-activated, heat-regulated, universal influenza vaccine

## Abstract

Vaccination with transiently activated replication-competent controlled herpesviruses (RCCVs) expressing influenza A virus hemagglutinins broadly protects mice against lethal influenza virus challenges. The non-replicating RCCVs can be activated to transiently replicate with high efficiency. Activation involves a brief heat treatment to the epidermal administration site in the presence of a drug. The drug co-control is intended as a block to inadvertent reactivation in the nervous system and, secondarily, viremia under adverse conditions. While the broad protective effects observed raise an expectation that RCCVs may be developed as universal flu vaccines, the need for administering a co-activating drug may dampen enthusiasm for such a development. To replace the drug co-control, we isolated keratin gene promoters that were active in skin cells but inactive in nerve cells and other cells in vitro. In a mouse model of lethal central nervous system (CNS) infection, the administration of a recombinant that had the promoter of the infected cell protein 8 (ICP8) gene of a wild-type herpes simplex virus 1 (HSV-1) strain replaced by a keratin promoter did not result in any clinical signs, even at doses of 500 times wild-type virus LD_50_. Replication of the recombinant was undetectable in brain homogenates. Second-generation RCCVs expressing a subtype H1 hemagglutinin (HA) were generated in which the infected cell protein 4 (ICP4) genes were controlled by a heat switch and the ICP8 gene by the keratin promoter. In mice, these RCCVs replicated efficiently and in a heat-controlled fashion in the epidermal administration site. Immunization with the activated RCCVs induced robust neutralizing antibody responses against influenza viruses and protected against heterologous and cross-group influenza virus challenges.

## 1. Introduction

We hypothesized that a recombinant virus capable of replicating in a controlled fashion with essentially the same efficiency as the virulent wild-type virus from which it has been derived should elicit a more potent, as well as a more balanced, immune response than a replication-defective or inactivated comparison vaccine or a subunit vaccine [1,2]. This hypothesis was in part based on the rational expectation that such an efficiently replicating recombinant virus would produce a particularly strong inflammatory response that should result in potent activation of the innate immune system and, consequently, in strong and lasting B and T cell responses. It was also supported by studies showing that replication-attenuated viruses induce superior and more balanced immune responses to themselves, as well as to heterologous antigens expressed from them, than corresponding replication-defective viruses [3,4,5].

The safety of such a recombinant virus would not come from attenuation but would be achieved by subjecting one or more replication-essential genes of a wild-type virus to the control of a tight gene switch that could be activated at will at a chosen inoculation site to trigger efficient but limited viral replication. We refer to such a regulated recombinant as a “replication-competent controlled virus” (RCCV). To usefully deploy a gene switch, the virus to serve as the backbone of an RCCV had to be a DNA virus (or, possibly, a retrovirus) that relies on the host cell for the transcription of its genes. We suggested that a wild-type alphaherpesvirus, in particular an HSV (i.e., HSV-1 or HSV-2), would be a good choice [1]. HSVs contain a large double-stranded DNA genome (>150 kbp) [6,7]. Genome stability is maintained by a high-fidelity DNA polymerase. The viruses remain episomal, alleviating concerns about untoward effects on the host cell genome. They cause only moderate morbidity and relatively rare mortality. While other cells are also productively infected, the primary targets of productive infection by HSVs are epithelial cells (in particular keratinocytes) in the skin and in mucosal membranes, cells which are lysed efficiently by the viruses. Of practical importance is that the viruses are readily manipulated, tolerate sizeable insertions of heterologous DNA, and support the expression of heterologous genes [8]. A valid concern with the employment of an HSV as the backbone virus is that it can infect nerve cells and remain present in a latent form. Reactivation can occur in response to a variety of stressors. A secondary concern is viremia which occurs rarely but to which immunocompromised subjects may be particularly susceptible. Therefore, the mechanism used to control an RCCV vaccine must be capable of preventing significant replication in neural cells and, to the extent possible, in all other cells except the epithelial cells to which the vaccine is to be administered.

The RCCVs that we developed previously employed a dual-activated, tight gene switch [9] that is activated by heat in the presence of an antiprogestin (AP) such as ulipristal or mifepristone but not by heat alone or by AP alone. Heat is a particularly suitable co-activator for an RCCV vaccine that is administered to the skin. Heat can be directed readily to a desired skin area. Hence, the activation of the vaccine can be confined to a narrow region that includes the administration site. Replication of an RCCV in the administration site may be adequately controlled by a gene switch that is activated by heat alone (i.e., a heat shock protein (HSP) gene promoter). However, such a gene switch may be activated inadvertently under adverse conditions (e.g., fever) which could result in systemic replication of the RCCV or reactivation in latently infected neural cells. To avoid such accidental replication or reactivation, we employed a dual-activated gene switch that is co-controlled by a drug. RCCVs containing this gene switch cannot replicate unless the drug is present. We opted for the APs ulipristal and mifepristone as the co-activating drugs because they are approved drugs and are essentially only used sporadically and in a single dose. Ulipristal and mifepristone have been used for emergency contraception or early pregnancy termination, respectively, for many years and have excellent safety records in these indications. Furthermore, the approval of ulipristal, mifepristone, and related drugs in other indications appears unlikely.

We tested our RCCVs extensively in vitro [10]. After activation, i.e., after heat treatment of infected cells in the presence of ulipristal (or mifepristone in early experiments), the recombinants transiently replicated with an efficiency approximating that of the backbone virus. Activation in vitro and in mouse skin was dependent on both heat and AP being present simultaneously. No replication was evident in the absence of activation treatment. The potential utility of the RCCVs as anti-herpetic vaccines was explored using a well-established mouse footpad lethal challenge model [11]. The activated RCCVs were far more efficacious in protecting against a lethal challenge by a wild-type HSV-1 strain than not-activated RCCVs or a replication-defective comparison virus. In a recent study, RCCVs expressing a full-length influenza virus subtype H1 or H3 HAs were investigated as potential influenza vaccines [12]. The RCCVs were applied to mice epidermally (to footpads), and the challenge influenza viruses were administered intranasally. The vaccines broadly protected against heterologous (intrasubtype) and cross-group challenges. No significant weight loss occurred after the challenges, and no signs of distress/disease were noted. Protective responses against an influenza B virus were also observed in animals vaccinated with RCCVs expressing HAs of influenza A subtype H1 and/or H3. Passive immunization experiments indicated that protection was at least in part antibody-mediated. To our knowledge, no other HA-directed vaccine has been capable of inducing similarly broad functional immune responses.

Exposure to influenza viruses and HSV-1 already occurs at an early age. Therefore, vaccination of young children against these pathogens may be indicated. This may be particularly beneficial in the case of influenza where it may be desirable to avoid imprinting by early influenza virus infection [13]. The administration of a drug (i.e., an AP) to young children for vaccination purposes may be considered undesirable or even unacceptable. In addition, the administration of a vaccine that needs to be co-activated by an AP may be contraindicated in women of reproductive age. We describe herein second-generation RCCVs that are similarly safe and potent influenza vaccines as the RCCVs we had studied previously but do not require the administration of a drug for activation. We also re-assess the importance of heat activation or co-activation.

## 2. Materials and Methods

### 2.1. Cells and Viruses

Rabbit skin (RS) cells were a gift from E. Wagner and were propagated in Eagle’s Minimal Essential Medium (MEM) (Life Technologies, Thermo Fisher Scientific, Waltham, MA, USA) supplemented with 5% heat-inactivated calf serum (Atlanta Biologicals, Lawrenceville, GA, USA) and 2 mM L-glutamine. Vero cells obtained from the American Type Culture Collection (ATCC, Manassas, VA, USA) and Vero-derived E5 cells [14] provided by N. DeLuca were propagated in Dulbecco’s Modified Eagle’s Medium (DMEM) with 10% heat-inactivated fetal bovine serum (FBS). HEK293T and HaCaT cells (ATCC) were grown in DMEM, Neuro-2a cells (ATCC) in MEM, and SH-SY5Y cells (ATCC) in a 1:1 mixture of MEM and F12 medium, the media supplemented with 10% heat-inactivated FBS, 2 mM L-glutamine, and penicillin/streptomycin. Stocks of RCCV HSV-GS19 were propagated on E5 cells and stocks of RCCVs HSV-GS19/mKRT1, HSV-GS61, HSV-GS62, HSV-GS63, and recombinants 17syn+/mKRT1 and HSV-GC9 on HEK293T cells. The medium was supplemented with 10 nM ulipristal (for HSV-GS19 and HSV-GS19/mKRT1 only), and infected cultures were subjected to daily heat treatment at 43.5 °C for 30 min for 3 successive days (all except 17syn+/mKRT1 and HSV-GC9). Stocks of recombinants HSV-GC1 and HSV-GC2 were produced in V17gH cells and stocks of HSV-GC3 in V17gH cells previously transfected with ICP8 expression construct pICP8 [10]. Influenza virus strain A/Fort Monmouth/1/1947(H1N1) (FM47) was procured from BEI Resources (Manassas, VA, USA), and strain A/Hong Kong/4801/2014(H3N2; mouse-adapted) (HK14) was a gift of E. B. Tarbet. Influenza virus strains were propagated on Madin–Darby canine kidney (MDCK) cells (ATCC) cultured in DMEM supplemented with 10% heat-inactivated horse serum, as previously described [15]. Influenza virus stocks were titrated using 50% tissue culture infective dose (TCID_50_) assays in MDCK cells. All cells were cultured at 37 °C under 5% CO_2_. HSV-1 strain 17syn+ was obtained from J. Stevens. Stocks were propagated in Vero or E5 cells.

### 2.2. Chemical Reagents

Ulipristal acetate (USP grade) was procured from D-Innovation Pharmaceutical Inc., Chengdu, China.

### 2.3. Construction of gH-Complementing Cell Line V17gH

Because the HSV-1 glycoprotein H (gH) is an essential protein and required for cell entry and spread, a complementing cell line was created in order to facilitate the construction of the HSV-GC1-3 deletion viruses and to allow efficient propagation of viral stocks. The Sleeping Beauty transposon system was used to efficiently introduce the HSV-1 gH gene into Vero cells. The gH gene from HSV-1 strain 17syn+ containing the native promoter and coding region (nt 43,824-46,581; Genbank NC_001806) was synthesized by Addgene (Watertown, MA, USA) with SfiI restriction sites at the 5′ and 3′ ends of the cloning cassette. This cassette was cloned into plasmid pSBtet-RN (Addgene plasmid #60506) containing the Sleeping Beauty transposon elements to generate plasmid pSBHSV17gH. Cultures of Vero cells in 60 mm dishes were transfected at 80% confluency with 7.5 µL TransIT-X2 (Mirus Bio, Madison, WI, USA) complexed with pSBHSV17gH alongside pCMV(CAT)T7-SB100 (a plasmid containing the transposase) using 2.5 µg DNA at a 9:1 plasmid mass ratio or, alternatively, were mock-transfected (reagent without DNA). Transformed cells could be identified by red fluorescent protein (RFP) expression. RFP signal was observed 24 h after transfection using an EVOS FL Cell Imaging System (Thermo Fisher Scientific). Cells were then subjected to selection with G418 (1 mg/mL G418S; ForMedium, Norfolk, UK), and cell line V17gH expressing gH was isolated.

### 2.4. Isolation and Characterization of Keratin 1 (KRT1) Promoters

Mouse promoter and 5′ untranslated (UTR) sequences were PCR-amplified from mouse genomic DNA (Promega, Madison, WI, USA) using a standard protocol. Promoter sequences lie upstream from the start of transcription site (−), and RNA leader (5′ UTR) sequences begin at the start of transcription site (+). PCR amplification employed either primers mKRT1F1 (5′-CTGACTGGCTTTAGCCCCTT-3′) and mKRT1R1 (5′-GCCTTAGAGAGAGGTGAGAGC-3′) or primers mKRT1F2 (5′-GCCACAAAACACTTTCAGGTACATA-3′) and mKRT1R2 (5′-TGATGCCTTAGAGAGAGGTGA-3′). The amplified mKRT1 segments were then subcloned into vector pGL4.16 (Promega) which contains a promoter-less luciferase reporter gene, luc2CP, from Photinus pyralis. To achieve this, the amplified mKRT1 DNAs were further amplified using forward and reverse primers containing KpnI and BamHI recognition sequences, respectively. The re-amplified DNAs were digested with KpnI and BamHI, and the fragments were gel-purified and then ligated into KpnI/BglII-double-digested pGL4.16. Following transformation, colonies were picked and expanded, and inserts were subjected to nucleotide sequence analysis. Clone mKRT1 1.2 contains mKRT1 sequences from position −993 to position +56 and clone mKRT1 2.3 mKRT1 sequences from position −1026 to position +60. Likewise, a short and a long segment of the human KRT1 promoter was amplified from human genomic DNA (Promega) using primer pairs hKRT1ShortF (5′-TTCTATTGCTGGTGTCTGTCTC-3′) and hKRT1ShortR (5′-GGAGCAAGGTAGAGTAAGGGAA-3′) for the short segment and hKRT1LongF (5′-GCAGCCGAAGGATTTTAGTGC-3′) and hKRT1LongR (5′-AGGAGCAAGGTAGAGTAAGGGA-3′) for the long segment. The PCR products were further amplified using forward and reverse primers also containing KpnI and BamHI recognition sequences, respectively. The amplification products were double-digested with KpnI and BamHI and ligated to KpnI/BglII-double-digested vector pGL4.16. Following transformation, colonies were picked and expanded, and inserts were subjected to nucleotide sequence analysis. Clone hKRT1 L1 contains hKRT1 sequences from position −2001 to position +39 and clone hKRT1 S3 hKRT1 sequences from position −724 to position +39. The ability of the KRT1 promoters to drive transcription from the functionally linked luciferase reporter genes was assessed in several cell types. Since expression was to be compared between different cell types which may differ in transfectability and general transcriptional activity, the different KRT1 constructs were co-transfected with a construct that contained a β-galactosidase reporter gene controlled by the ubiquitously active ROSA26 promoter (pDRIVE-mROSA; InvivoGen Corp, San Diego, CA, USA). Transfection of subconfluent cultures employed a standard lipofectamine procedure (Lipofectamine 2000; Life Technologies). The activities of the KRT1 promoters were expressed as ratios of relative luciferase to β-galactosidase activities. Luciferase activity was measured using the Dual Glo Luciferase Assay System (Promega) and β-galactosidase activity using the Beta-Glo Assay System (Promega).

### 2.5. Construction of Recombinant Viruses

RCCV HSV-GS19/mKRT1 was derived from HSV-GS19 [12]. It contains a heat- and AP-responsive gene switch that controls ICP4 expression. The recombinant also carries an expressible HA gene of influenza virus strain A/California/07/2009(H1N1) (CA09). Furthermore, an mKRT1 promoter controls the expression of its ICP8 gene. To generate recombinant HSV-GS19/mKRT1, HEK293T cells were co-transfected with plasmid pBS-KS:mKRT1-ICP8 and purified HSV-GS19 virion DNA by calcium phosphate precipitation [8]. After the addition of 10 ng/mL ulipristal to the medium, the transfected cells were exposed to 43.5 °C for 30 min and then incubated at 37 °C. Subsequently, on days 2 and 3, the cells were again incubated at 43.5 °C for 30 min and then returned to 37 °C. Several plaques were picked and amplified on 96-well plates of HEK293T cells in medium supplemented with ulipristal. The plates were incubated at 43.5 °C for 30 min 1 h after infection and then incubated at 37 °C. Subsequently, on days 2 and 3, the plates were also shifted to 43.5 °C for 30 min and then returned to 37 °C. After the wells showed 90–100% cytopathic effect (CPE), the plates were dot-blotted, and the dot blot membrane was hybridized with a ^32^P-labeled DNA probe prepared by labeling the mKRT1 promoter segment by random-hexamer priming. A positive well was re-plaqued and re-probed several times and was verified by PCR and sequence analysis to have lost the GAL4 promoter at ICP8 and to contain the mKRT1 promoter in its place. To construct plasmid pBS-KS:mKRT1-ICP8, plasmid pmKRT1 1.2 containing an mKRT1 promoter was subjected to PCR amplification using primers mKRT1-AatIIF (5′-TAGTGACGTCCTGACTGGCTTTAGCCCCTT-3′) and mKRT1-AatIIR (5′-AAAGACGTCGCCTTAGAGAGAGGTGAGAGCAAAGACA-3′). The amplified fragment containing mKRT1 sequences from position −995 to position +56 was digested with AatII and gel-purified. For the vector, plasmid pBS-KS:ICP8∆promoter containing HSV-1 sequences flanking the ICP8 promoter [10] was digested with AatII. The resulting 4.59 kbp fragment was gel-purified. Ligation of the latter two DNA fragments placed the mKRT1 promoter in front of the ICP8 transcription start site. Subsequent to transformation, several colonies were expanded, and plasmid DNAs were subjected to restriction and then nucleotide sequence analysis to identify pBS-KS:mKRT1-ICP8. The correct orientation of the promoter was also verified by HindIII digestion. The mKRT1 sequence contains a HindIII site at position −917, and the polylinker of pBS-KS:ICP8∆promoter also contains a HindIII site. Recombinant 17syn+/mKRT1 was derived from wild-type HSV-1 strain 17syn+. An mKRT1 promoter controls the expression of its ICP8 gene. To produce recombinant 17syn+/mKRT1, HEK293T cells were co-transfected with plasmid pBS-KS:mKRT1-ICP8 and purified 17syn+ virion DNA by calcium phosphate precipitation. Several plaques were picked and amplified on 96-well plates of HEK293T cells. After the wells showed 90–100% CPE, the plates were dot-blotted, and the dot blot membrane was hybridized with a ^32^P-labeled DNA probe prepared by labeling the mKRT1 promoter segment. A positive well was re-plaqued and re-probed several times and was verified to have lost the ICP8 promoter and to contain the mKRT1 promoter in its place by PCR and sequence analysis. Recombinant HSV-GC9 was derived from 17syn+/mKRT1. It contains an mKRT1 promoter-controlled ICP8 gene. Furthermore, it carries the HA gene of influenza virus strain CA09(H1N1) driven by a cytomegalovirus immediate-early (CMV IE) promoter. Plasmid pIN:37/38-Cal/07/HA [12] was co-transfected with purified 17syn+/mKRT1 virion DNA into HEK293T cells by calcium phosphate precipitation. Plasmid pIN:37/38-Cal/07/HA contains the expressible CA09 HA gene in between sequences from the UL37/38 intergenic region of 17syn+ (nt 83,603-84,417). Picking and amplification of plaques, screening, and plaque purification were performed essentially as described for 17syn+/mKRT1, except that dot blots were hybridized with a ^32^P-labeled DNA probe prepared by labeling the HA gene. One recombinant clone was verified by Southern blot analysis as well as by sequencing of the HA insert and ~200 bp of the flanking sequence on each side of the insert. The ability of this recombinant to produce the HA protein was confirmed by enzyme-linked immunosorbent assay (ELISA) on lysates of infected cells. RCCVs HSV-GS61 and HSV-GS62 were derived from HSV-GS19/mKRT1. They contain a heat-responsive gene switch (different in the two recombinants) that controls ICP4 expression. The recombinants also carry an expressible HA gene of CA09(H1N1). Furthermore, an mKRT1 promoter controls the expression of the ICP8 gene. Recombination plasmid pINTA was described in ref. [10]. It contains, inserted into sequences of the UL43/44 intergenic region of strain 17syn+, a promoter assembly comprising an HSP70B (HSPA7) promoter and a GAL4 promoter. This assembly is functionally linked to a gene for chimeric transactivator (TA) GLP65 comprising a GAL4 DNA-binding domain, a truncated ligand-binding domain from a progesterone receptor (that binds APs such as ulipristal), and an activation domain. The sequences in pINTA that encode the progesterone receptor ligand-binding domain of GLP65 were removed using the Q5^®^ Site-Directed Mutagenesis Kit from New England BioLabs (Ipswich, MA, USA) and oligonucleotides DelGLP65F (5′-GGGTCGACGCCCATGGAA-3′) and DelGLP65R (5′-CTGGTCGACACCCGGGAATTC-3′). The resulting plasmid was termed pINTAΔPRL-BD. The GAL4 promoter of the promoter assembly driving TA expression was deleted by digesting pINTAΔPRL-BD with Sgf1 [9] and self-ligating the larger fragment. The resulting plasmid was named pINTAΔPRL-BDΔGAL4. To construct plasmid pINTAΔPRL-BDΔGAL4 Hairpin 4, phosphorylated oligonucleotides Hairpin4F (5′CCGGGATCCAACAACAACAACAACCCTGCGGTCCACCACGGCCGATATCACGGCCGTGGTGGACCGCAGGGCAACAACAACAACAACGGAT-3′) and Hairpin4R (5′CCGTTGTTGTTGTTGTTGCCCTGCGGTCCACCACGGCCGTGATATCGGCCGTGGTGGACCGCAGGGTTGTTGTTGTTGTTGGATCCCGGAT-3′) were annealed and inserted into Sgf1-digested plasmid pINTAΔPRL-BDΔGAL4. Plasmids pINTAΔPRL-BDΔGAL4 Hairpin 1, 2, or 3 containing hairpin sequences 1, 2, or 3 were prepared analogously using phosphorylated oligonucleotides Hairpin1F (5′-CCGGGATCCAACAACAACAACAAGTCCACCACGGCCGATATCACGGCCGTGGTGGACCAACAACAACAACAACGGAT-3′) and Hairpin1R (5′-CCGTTGTTGTTGTTGTTGGTCCACCACGGCCGTGATATCGGCCGTGGTGGACTTGTTGTTGTTGTTGGATCCCGGAT-3′); Hairpin2F (5′-CCGGGATCCAACAACAACAACAAGAGTCCACCACGGCCGATATCACGGCCGTGGTGGACTCCAACAACAACAACAACGGAT-3′) and Hairpin2R (5′-CCGTTGTTGTTGTTGTTGGAGTCCACCACGGCCGTGATATCGGCCGTGGTGGACTCTTGTTGTTGTTGTTGGATCCCGGAT-3′); and Hairpin3F (5′-CCGGGATCCAACAACAACAACAAGCGGTCCACCACGGCCGATATCACGGCCGTGGTGGACCGCCAACAACAACAACAACGGAT-3′) and Hairpin3R (5′-CCGTTGTTGTTGTTGTTGGCGGTCCACCACGGCCGTGATATCGGCCGTGGTGGACCGCTTGTTGTTGTTGTTGGATCCCGGAT-3′). To produce recombinant HSV-GS61, HEK293T cells were co-transfected with plasmid pINTAΔPRL-BDΔGAL4 and purified HSV-19/mKRT1 virion DNA by calcium phosphate precipitation. The transfected cells were exposed to 43.5 °C for 30 min and then incubated at 37 °C. Subsequently, on days 2 and 3, the cells were again incubated at 43.5 °C for 30 min and then returned to 37 °C. Several plaques were picked and amplified on 96-well plates of HEK293T cells (with daily heat treatment). After several rounds of re-plaquing, the isolates were verified to contain the modified promoter–TA gene sequences by PCR and nucleotide sequence analysis. One of these isolates was termed HSV-GS61. Recombinant HSV-GS62 was produced by the same procedure, except that co-transfection was with plasmid pINTAΔPRL-BDΔGAL4 Hairpin 4 and HSV-19/mKRT1 virion DNA. RCCV HSV-GS63 was also derived from HSV-GS19/mKRT1. Its ICP4 genes are controlled by HSP70B (HSPA7) promoters and its ICP8 gene by an mKRT1 promoter. The recombinant also carries an expressible HA gene of influenza virus strain CA09(H1N1). The heat- and AP-responsive gene switch of HSV-GS19/mKRT1 is retained but is non-functional because the recombinant lacks any GAL4 promoter-controlled gene. To produce RCCV HSV-GS63, HEK293T cells were co-transfected with plasmid pBS-KS:HSP70-ICP4 and purified HSV-19/mKRT1 virion DNA by calcium phosphate precipitation. Picking and amplification of plaques were performed essentially as described for HSV-GS61. After the wells showed 90–100% CPE, the plates were dot-blotted, and the dot blot membrane was hybridized with a ^32^P-labeled HSP70B promoter probe. A strongly positive well was re-plaqued and re-probed several times, and the isolated recombinant was verified by PCR and nucleotide sequence analysis to have lost the GAL4 promoters upstream from the ICP4 genes and to contain HSP70B promoters in their place. Plasmid pBS-KS:HSP70-ICP4 was constructed by PCR amplification of a short HSP70B promoter segment (about 480 bp in length) from plasmid pHsp70-fluc (described in ref. [9]), using primers Hsp70promAatIIF (5′-ATTCGACGTCTCGCCTCAGGGATCCGACCT-3′) and Hsp70promHindIIIR (5′-TCTAGAGTCGACCTGCAGGCATGCAAGCTTCTTGT-3′), digestion of the amplified fragment with AatII and HindIII, and ligation of the digested fragment to AatII/HindIII-digested vector pBS-KS:ICP4∆promoter (described in ref. [10]). Recombinant HSV-GC1 was derived from wild-type HSV-1 strain 17-syn+ and contains a deletion in the gH gene. In order to delete the gH gene, flanking sequences to the desired deletion (nt 43,824-46,581) (left recombination arm: nt 43,650-43,823; right recombination arm: nt 46,582-46,821; synthesized by Addgene) were subcloned into the EcoRV site of plasmid pBS-SK+. V17gH cells were co-transfected with the latter plasmid (p17∆gH) and purified 17syn+ virion DNA by calcium phosphate precipitation. Picking and amplification of plaques, screening, and plaque purification (in V17gH cells) were performed essentially as described for 17syn+/mKRT1, except that dot blots were performed in duplicate and were hybridized with ^32^P-labeled DNA probes prepared by labeling the HSV-1 gH fragment (nt 43,824-46,581) and the HSV-1 DNA polymerase gene, respectively. Cell lysates from 3 wells that were negative for the gH gene but positive for the HSV-1 DNA polymerase gene were re-plaqued and re-probed 5 times. One recombinant, designated HSV-GC1, was characterized by nucleotide sequencing across the deleted region and Southern blot analysis as well as tested for its inability to spread and form plaques on Vero cells. Recombinant HSV-GC2 was derived from HSV-GC1. It contains a deletion in the gH gene. Furthermore, it carries the HA gene of CA09(H1N1) driven by the CMV IE promoter. Plasmid pIN:37/38-Cal/07/HA [12] was co-transfected with purified HSV-GC1 virion DNA into V17gH cells by calcium phosphate precipitation. Picking and amplification of plaques, screening, and plaque purification were performed essentially as described for HSV-GC9. One recombinant was verified by Southern blot analysis as well as by sequencing of the HA insert and ~200 bp of the flanking sequence on each side of the insert. The ability of this recombinant to produce the HA protein was confirmed by ELISA on lysates of infected cells. Recombinant HSV-GC3 was derived from HSV-GC2. Its ICP8 gene is controlled by an mKRT1 promoter. To produce recombinant HSV-GC3, V17gH cells previously transfected with expression construct pICP8 were co-transfected with plasmid pBS-KS:mKRT1-ICP8 and purified HSV-GC2 virion DNA by calcium phosphate precipitation. Picking and amplification of plaques, screening, and plaque purification were performed essentially as described for 17syn+/mKRT1, except that pICP8-transfected V17gH cells were employed. A selected recombinant was verified to have lost the ICP8 promoter and to contain the mKRT1 promoter in its place by PCR and nucleotide sequence analysis.

A summary description of all recombinants employed in the present study and their essential features is provided in Table 1.

### 2.6. Evaluation of the Activity and Heat Regulation of Recombination Plasmids Containing Modified Promoter–Transactivator Cassettes

Subconfluent cultures of Vero cells (25,000 cells per well of a 96-well plate) were co-transfected with a transactivator plasmid (pINTAΔPRL-BDΔGAL4 or pINTAΔPRL-BDΔGAL4 Hairpin 1, 2, 3, or 4), a reporter plasmid (pGAL4-fLuc; described in ref. [9]), a plasmid containing a Renilla luciferase gene driven by a CMV IE promoter for normalization (pRL-CMV; Promega), and pBlueScript II DNA to equalize total DNA amounts transfected using Lipofectamine 2000, according to the manufacturer’s protocol. Plates containing transfected cultures were either kept at 37 °C or subjected to a 30 min heat treatment at 43.5 °C by partial immersion in a temperature-controlled water bath. Six hours later, luciferase activities were determined using the Dual Glo Luciferase Assay System.

### 2.7. Statistical Analyses

Where appropriate, data are presented as mean values with standard deviation. The statistical analyses were performed using the Statistical Program for Social Sciences version 25 (IBM Corp.; Armonk, NY, USA). Kaplan–Meier survival curves were analyzed for significance using the log-rank test. Kolmogorov–Smirnov normality tests were used to evaluate whether the data followed a normal distribution. Parametric data from 3 or more groups were analyzed using one-way analysis of variance (ANOVA) followed by Bonferroni’s multiple comparison test while non-parametric data were tested using the Kruskal–Wallis test followed by Mann–Whitney *U*-test for post hoc group comparisons. The Student’s *t*-test and Mann–Whitney *U*-test were used to compare two groups with normally and non-normally distributed data, respectively. No data sets that were analyzed for statistical significance varied in sample size by more than 3-fold. The criterion for significance was set at 0.05.

### 2.8. Other Methods

Single-step growth experiments, immunizations, determinations of titers of RCCVs in mouse feet or brains, or challenge viruses in mouse lungs, blood collection, and microneutralization assays as well as HA ELISA tests as well as statistical analyses were performed as described in ref. [12]. Immunized and control mice were challenged by intranasal administration of 20 TCID_50_ of influenza virus strain FM47 or 10 TCID_50_ of influenza virus strain HK14.

Conclusions reached from animal experiments were supported by results from at least two independent experiments and redundancies within individual experiments.

## 3. Results

### 3.1. First-Generation RCCVs

**Figure 1:**
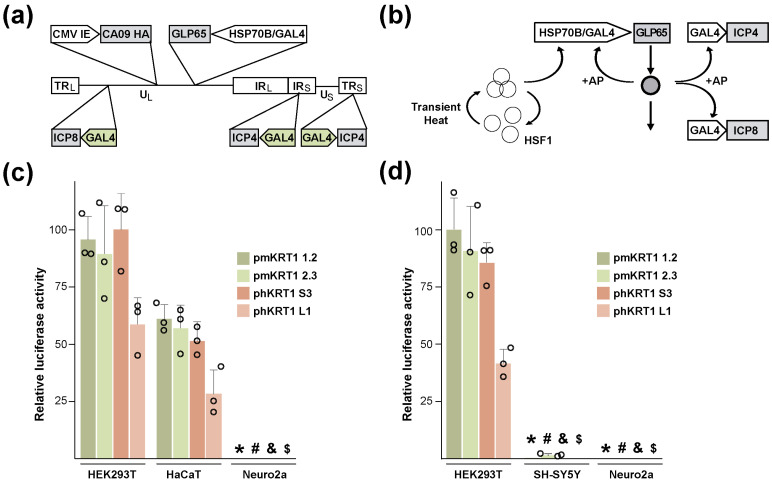
RCCV structure, dually-controlled gene switch, and characterization of KRT1 constructs. (**a**) Diagrammatic representation of the genome structure of HSV-1-derived RCCV HSV-GS19. The RCCV contains a CMV immediate early-promoter (CMV IE)-driven full-length HA gene of influenza virus strain A/California/07/2009 (CA09 HA) inserted into the intergenic region between UL37 and UL38. A gene for transactivator GLP65 functionally linked to a promoter assembly comprising a human HSP70B promoter (HSP70B) and a GAL-responsive promoter (GAL4) is inserted into the intergenic region between UL43 and UL44. The promoters of the replication-essential HSV-1 genes ICP4 and ICP8 are replaced with GAL4 promoters. TR_L_, TR_S_: long and short terminal repeats; U_L_, U_S_: long and short unique regions; IR_L_, IR_S_: long and short internal repeats. (**b**) Dually-responsive gene switch in HSV-GS19: a promoter assembly comprising an HSP70B promoter (HSP70B) and a GAL4-responsive promoter (GAL4) controls a gene for AP-activated transactivator GLP65. The replication-essential ICP4 and ICP8 genes are controlled by GAL4 promoters. Heat treatment of a cell infected with HSV-GS19 transiently activates the cellular heat shock factor (HSF1) that then transactivates the GLP65 gene. Newly synthesized, inactive GLP65 molecules are activated when bound by an AP. Activated GLP65 transactivates the GAL4 promoter-controlled ICP4 and ICP8 genes as well as its own gene. (**c**,**d**) Characterization of KRT1 promoters: Triplicate, subconfluent cultures (25,000 cells per well; 96-well plates) of HEK293T, HaCaT, and Neuro-2a cells (**c**) or HEK293T, SH-SY5Y, and Neuro-2a cells (**d**) were co-transfected with 159 ng of plasmid pmKRT1 1.2, pmKRT1 2.3, phKRT1 S3, phKRT1 L1, or pGL4.16 and 1 ng of plasmid pDRIVE-mROSA using a standard lipofectamine transfection protocol. Relative activities of the KRT1 promoters expressed as ratios of luciferase activities (after subtraction of pGL4.16 background activity) to β-galactosidase activities were determined one day after transfection. Relative activities (actual and mean values) and standard deviations from representative experiments are shown. *p* ≤ 0.05, comparing to HEK293T or HaCaT cells transfected with plasmid pmKRT1 1.2 (*), pmKRT1 2.3 (#), phKRT1 S3 (&), or phKRT1 L1 ($).

RCCV HSV-GS19 served as a benchmark for the present study. This RCCV (shown diagrammatically in Figure 1a) and its vaccine use were described previously [12]. HSV-GS19 was derived from virulent HSV-1 strain 17syn+. It contains inserted in the UL43/44 intergenic region a gene for chimeric AP-activated transactivator GLP65 that comprises a GAL4 DNA-binding domain, a truncated ligand-binding domain from a progesterone receptor, and an activation domain. The expression of the GLP65 gene is controlled by a promoter assembly that combines a human HSP70B (HSPA7) gene promoter functioning essentially as an on–off switch and a GLP65-responsive promoter (GAL4). The promoters of the replication-essential genes encoding ICP 4 (both copies) and ICP8 are replaced with GAL4 promoters. A full-length HA gene from influenza virus strain CA09(H1N1) that is controlled by a CMV IE promoter is inserted in the UL37/38 intergenic region. After activation of the recombinant, infected cells synthesize the HA at a robust rate [12]. The operation of the dually controlled gene switch is illustrated in Figure 1b. When a cell infected with recombinant HSV-GS19 is subjected to a transient heat treatment, the cellular transcription factor HSF1 (heat shock factor 1) is transiently activated and mediates the expression of the HSP70B promoter-driven GLP65 gene. GLP65 is synthesized in an inactive form. When present, AP binds to and activates GLP65. Activated GLP65 then transactivates the GAL4 promoter-driven genes, i.e., the ICP4 and ICP8 genes, as well as its own gene (maintaining GLP65 at a functional level). The gene switch is inactivated by the withdrawal of the AP or upon lysis of the infected cell.

### 3.2. Characterization of KRT1 Promoters

The AP co-control of the RCCV was intended as a mechanism for precluding virus reactivation in latently infected neural cells as well as unwanted systemic replication under adverse conditions. We hypothesized that it may be possible to prevent reactivation in latently infected neural cells and, more generally, replication of an RCCV in cells other than skin cells by subjecting a replication-essential gene of the virus to the control of a promoter that is essentially inactive in nerve cells and most other cells but is highly active in epithelial cells, in particular cells of the epidermis. If this could be achieved, the AP co-control would be rendered redundant. Database mining suggested that certain keratin genes exhibit the desired expression pattern. These included the human and mouse KRT1 and KRT10 genes as well as the mouse KRT77 gene. Fragments containing 993 or 1026 bp of 5′ nontranscribed sequence and 56 or 60 bp, respectively, of transcribed sequence of the mouse KRT1 gene or 724 bp of 5′ nontranscribed sequence and 39 bp of transcribed sequence of the human KRT1 gene were PCR-amplified from genomic DNA. A fragment containing 2001 bp of 5′ nontranscribed sequence and 39 bp of transcribed sequence of the human KRT1 gene was also obtained. These fragments were subcloned into the promoter-less vector pGL4.16 which comprises the luciferase gene luc2CP from Photinus pyralis. Constructs mKRT1 1.2 and mKRT1 2.3 contain mouse KRT1 promoter sequences of slightly different lengths (993 bp and 1026 bp). Constructs hKRT1 L1 and S3 harbor human KRT1 segments encompassing 2001 bp and 724 bp of 5′ untranscribed sequence, respectively. The activities of these constructs were assessed in different cell lines. To allow for comparisons across cell lines, the constructs were co-transfected with pDrive-mROSA, a plasmid carrying the E. coli LacZ gene that is controlled by the ubiquitously active ROSA26 promoter [16]. Results from co-transfection experiments in which the KRT1 promoters were tested in mouse neuroblastoma Neuro-2a cells, human neuroblastoma SH-SY5Y cells, human keratinocyte HaCaT cells, and human embryonic kidney HEK293T cells are shown in Figure 1c,d. The KRT1 promoters were active in HaCaT and HEK293T cells but essentially inactive in Neuro-2a and SH-SY5Y cells. In additional experiments, the KRT1 promoters were also found to be essentially inactive in Vero and HEK293 cells. Hence, the isolated KRT1 promoters appeared to exhibit the desired tissue selectivity. It is noted that a human KRT1 promoter was previously found to be active in HEK293T cells but not in HEK293 cells [17].

### 3.3. KRT1 Promoter Control Prevents Viral Replication in the CNS but Does Not Significantly Affect RCCV Vaccine Efficacy

**Figure 2:**
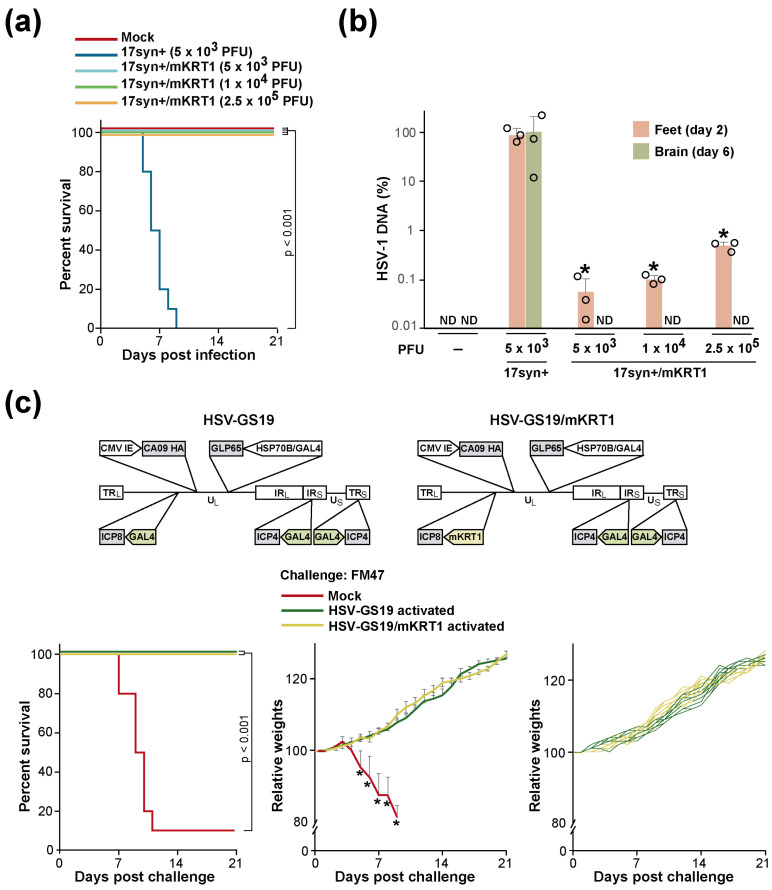
Absence of spread and replication of a KRT1 promoter-restricted HSV-1 recombinant in the CNS in vivo and vaccine efficacy of a KRT1 promoter-restricted RCCV. (**a**) Survival of mice inoculated with recombinant 17syn+/mKRT1. Groups (n = 10) of adult BALB/c mice were inoculated on the rear footpads with 5 × 10^3^ PFU/animal of HSV-1 wild-type strain 17syn+ or 5 × 10^3^, 1 × 10^4^, or 2.5 × 10^5^ PFU/animal of recombinant 17syn+/mKRT1, or vehicle (mock). The data are presented as percent survival for each treatment group. (**b**) Replication of virus in the feet and brains of BALB/c mice infected with 17syn+ or 17syn+/mKRT1 assessed by qPCR. DNA was isolated from mouse feet (2 days after inoculation) and brains (6 days after inoculation), and TaqMan PCR was performed using primers/probe specific for the HSV-1 DNA polymerase gene. Results are relative to quantities of HSV-1 DNA in brains infected with 17syn+ and are presented as mean values of relative genomes/mg tissue with standard deviations. A relative amount of 100 corresponded to 6.2 × 10^3^ genomes/mg tissue. ND: not detected. Sensitivity: 0.15 genomes/mg tissue. * *p* ≤ 0.05, comparing to mouse feet infected with 17syn+. (**c**) Vaccine efficacy of mKRT1 promoter-restricted RCCV HSV-GS19. The diagrammatic structures of HSV-GS19 and HSV-GS19/mKRT1 are shown on top. See Figure 1 for additional explanations. Groups (n = 10) of adult BALB/c mice were inoculated on their rear footpads with 2.5 × 10^5^ PFU/animal of RCCV HSV-GS19 or HSV-GS19/mKRT1, or vehicle (mock), and the RCCV-inoculated groups were subjected, 3 h later, to a 10 min heat treatment of their hindlimbs at 44.5 °C. Ulipristal (50 µg/kg body weight) was administered IP at the time of inoculation as well as on the following day. Three weeks after inoculation, the mice were immunized again, and, after three further weeks, all mice were challenged by intranasal administration of a lethal dose of heterologous influenza virus strain FM47(H1N1). Animals were observed daily, and weights were recorded. Left graph: survival (≤20% weight loss) after challenge; center graph: averaged relative weights of surviving animals after challenge. Weights are relative to weights on the day of the challenge. Relative values (down to the nadir in the case of the mock group comprising a survivor) and standard deviations are shown. * *p* ≤ 0.05, comparing to the HSV-GS19 or the HSV-GS19/mKRT1 group; right graph: relative weights after the challenge of all animals in the RCCV-immunized groups. Weights are relative to weights on the day of the challenge.

The mouse footpad lethal challenge model [18] was employed to functionally test whether subjecting a replication-essential viral gene to the control of a KRT1 promoter would reduce or prevent relevant virus replication in the CNS in vivo. The clinical endpoints in this model are indicative of severe CNS infection (bilateral hindlimb paralysis, inability to move when touched, or trembling). Recombinant 17syn+/mKRT1 was generated in which the endogenous promoter of the replication-essential ICP8 gene of wild-type virus 17syn+ was replaced with a mouse KRT1 promoter. The recombinant replicated efficiently in HEK293T cells and rabbit skin cells. Groups (n = 10) of adult BALB/c mice were inoculated on their slightly abraded rear footpads with 5 × 10^3^ plaque-forming units (PFU) per animal of wild-type HSV-1 strain 17syn+ (10 × LD_50_) or 5 × 10^3^, 1 × 10^4^, or 2.5 × 10^5^ PFU/animal of recombinant 17syn+/mKRT1, or vehicle (mock). The animals were followed for 21 days. All animals of the 17syn+ group but none of the 17syn+/mKRT1 groups or the mock-inoculated group reached the clinical endpoint (Figure 2a). No signs of distress or disease were observed in the latter groups. These results strongly suggested that 17syn+/mKRT1 was unable to traffic through the peripheral nervous system and cause encephalitis in the CNS. A second set of groups of animals that were similarly inoculated with strain 17syn+ or recombinant 17syn+/mKRT1 or were mock-inoculated were analyzed for the spread of the virus. Three animals of each group were sacrificed two days after inoculation, and three additional animals were sacrificed six days after inoculation; they were dissected, and DNA was extracted from inoculated feet and brains, respectively. Quantitative PCR analysis of these DNA samples using primers and a probe specific for the HSV-1 DNA polymerase gene indicated that recombinant 17syn+/mKRT1 replicated in the mouse feet (day 2 samples) (Figure 2b). Recombinant 17syn+/mKRT1 was undetectable in the mouse brains (day 6 samples), whereas substantial numbers of genomes of strain 17syn+ could be documented.

We next inquired whether an RCCV whose tropism was restricted by subjecting a replication-essential viral gene to mKRT1 control could still function as an effective influenza vaccine. Recombinant HSV-GS19/mKRT1 was constructed by replacing the GAL4 promoter upstream from the ICP8 gene in HSV-GS19 with an mKRT1 promoter. Thus, the resulting RCCV had ICP4 genes that were controlled by the heat- and AP-activated gene switch and an ICP8 gene whose expression was restricted by the mKRT1 promoter. Groups (n = 10) of adult BALB/c mice were inoculated on the slightly abraded plantar surfaces of their rear feet with 2.5 × 10^5^ PFU/animal of HSV-GS19 or HSV-GS19/mKRT1, or vehicle. All animals of RCCV-inoculated groups also received an intraperitoneal (IP) injection of 50 µg/kg of body weight of ulipristal at the time of inoculation and, again, on the subsequent day. Heat treatments (44.5 °C for 10 min) were administered to the hindlimbs of the animals 3 h after inoculation. Three weeks later, all mice were immunized (and RCCVs activated) again as before and, after a further three weeks, were challenged intranasally with a lethal dose of heterologous influenza virus strain FM47(H1N1). All challenged animals were observed daily, and weights were recorded for 3 wk. All animals that had been immunized with activated HSV-GS19 or HSV-GS19/mKRT1 were fully protected against the lethal challenge (Figure 2c, left graph). Furthermore, the immunized animals did not show any signs of illness and exhibited normal weight gain over the course of the observation period (Figure 2c, center and right graphs). It is noted that young adult (10–12 wk old) BALB/c mice are well known to continue gaining weight. Hence, the restriction of RCCV replication to the skin did not diminish vaccine efficacy (at least not against the heterologous influenza virus strain tested).

### 3.4. Engineering out the AP Co-Control from a KRT1 Promoter-Restricted RCCV

**Figure 3:**
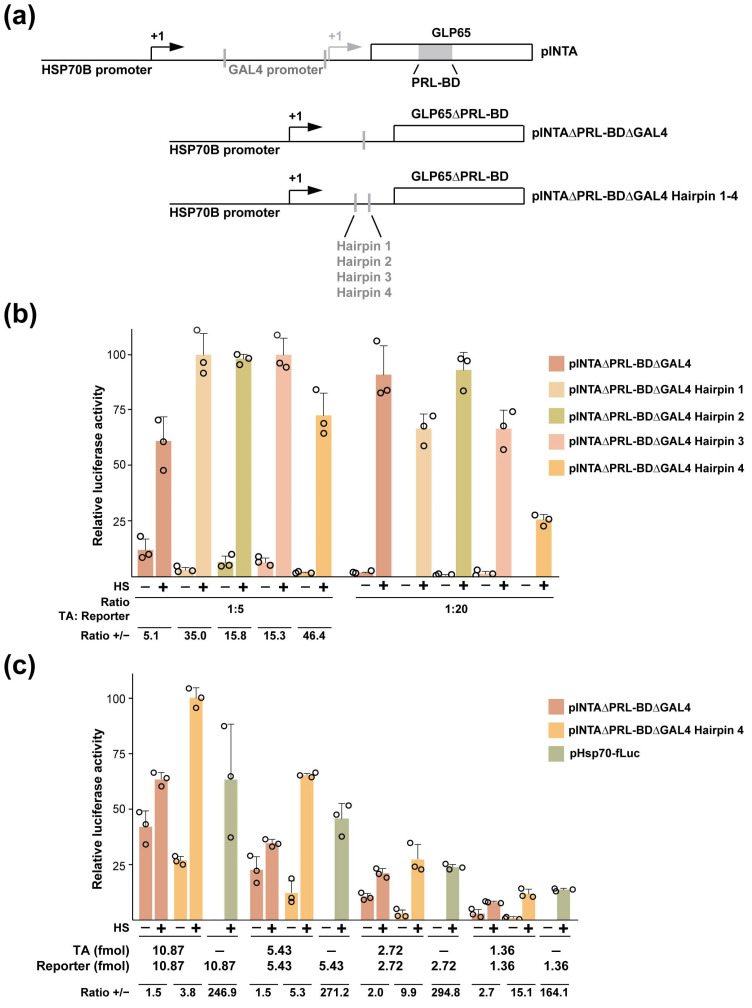
Modified promoter–transactivator cassettes and heat regulation conferred by them. (**a**) Diagrams of the structures of recombination plasmids (transactivator gene plasmids) are shown containing the original HSP70B/GAL4-GLP65 cassette present in HSV-GS19 (pINTA), a cassette lacking the GAL4 promoter and the sequences encoding the AP-binding domain of GLP65 (pINTAΔPRL-BDΔGAL4), and cassettes additionally containing different hairpin sequences inserted in the 5′ UTR of the modified GLP65 gene (pINTAΔPRL-BDΔGAL4 Hairpin 1-4). (**b**) Activities of the different promoter–transactivator cassettes at mass ratios of transactivator gene plasmid to reporter gene plasmid pGAL4-fLuc of 1:5 or 1:20 (amounts of reporter gene plasmid and total DNA kept constant). Two sets of triplicate, subconfluent cultures of Vero cells (25,000 cells per well; 96-well plates) were co-transfected with a transactivator gene plasmid, reporter gene plasmid pGAL4-fLuc, plasmid pRL-CMV (containing a CMV IE promoter-driven Renilla luciferase gene) for normalization, and plasmid pBluescript II using a standard lipofectamine transfection protocol. On the following day, one of the two sets of parallel cultures was subjected to a 30 min heat treatment at 43.5 °C. Six hours later, luciferase activities were determined. Data from a representative experiment are presented as normalized relative firefly luciferase activities (actual values and mean values with standard deviations). Shown below the graph are ratios of heat-induced and not-induced firefly luciferase activities. (**c**) Comparison of the activities of the transactivator cassettes of recombination plasmids pINTAΔPRL-BDΔGAL4 and pINTAΔPRL-BDΔGAL4 Hairpin 4 co-transfected with pGAL4-fLuc at a 1:1 molar ratio. Transactivator gene and reporter gene plasmids were transfected in decreasing amounts, as indicated in the graph. Cells transfected with plasmid pHsp70-fLuc were included for comparison. Total amounts of transfected DNA were kept constant by the addition of plasmid pBluescript II DNA. Results from a representative experiment performed as described under (**b**) are presented. Shown below the graph are ratios of heat-induced and not-induced firefly luciferase activities.

Our preferred approach for eliminating the AP co-control of ICP4 gene expression in RCCV HSV-GS19/mKRT1 was to replace the promoter assembly controlling GLP65 expression with a single HSP70B promoter and to delete the GLP65 gene sequences encoding the AP-binding domain. The presence of an uncontrolled instead of a controlled transactivator (TA) in the gene switch could be expected to result in an extended period of expression of the ICP4 genes after heat activation. However, this advantage may be obtained at the expense of a reduced stringency of heat regulation. Co-transfection experiments were carried out to find out whether the modified gene switch could still confer heat regulation on a target gene. Vero cells were co-transfected with construct pINTAΔPRL-BDΔGAL4, a recombination plasmid carrying an HSP70B promoter-driven gene for a constitutively active TA (i.e., AP-binding site-deleted GLP65) (Figure 3a) or constructs carrying further modified TA cassettes (discussed below; see also Figure 3a), a plasmid containing a GAL4 promoter-controlled firefly luciferase gene (target gene construct pGAL4-fLuc), and a plasmid expressing Renilla luciferase (pRL-CMV) for normalization. One day later, one of each pair of transfected cultures was subjected to a heat treatment. Dual luciferase assays were performed 6 h later. Different (mass) ratios of TA gene construct to target gene construct were tested (with amounts of total DNA and target gene construct kept constant). Reasonably stringent heat regulation of target gene expression was observed in cells transfected with construct pINTAΔPRL-BDΔGAL4 and pGAL4-fLuc at a 1:20 ratio (Figure 3b). However, significant unregulated target gene expression occurred at a 1:5 ratio of TA construct to target gene construct. Since an RCCV will contain one TA gene and one or two target genes, we conducted similar co-transfection experiments in which decreasing amounts of TA and target gene constructs were transfected, and a 1:1 molar ratio of TA and target gene constructs was maintained. Representative results from such an experiment are shown in Figure 3c. Substantial unregulated target gene expression was observed at the higher amounts of co-transfected TA and target genes. However, the stringency of heat regulation increased with decreasing amounts of co-transfected constructs. Assuming that cells in the administration site of a vaccine recipient will only be infected with a relatively low number of RCCV particles, an RCCV controlled by the modified gene switch may be stringently heat-regulated. However, as a precaution, we sought to improve the stringency of heat regulation by limiting TA expression. To achieve this, hairpin sequences with calculated thermal stabilities ranging from 35 to 50 kcal/mol [19] were introduced into the 5′ UTR of the TA gene (Figure 3a). We found that the inclusion of hairpin sequences reduced unregulated target gene expression (Figure 3b; best seen at the 1:5 ratio of TA to target gene constructs). Hairpin 4 appeared to be the most effective. At a 1:1 ratio of TA to target gene constructs, the TA containing hairpin 4 controlled target gene expression more stringently than the TA lacking the hairpin sequence, especially at low amounts of co-transfected constructs (Figure 3c). The stringency of regulation approached, but did not attain, that of direct HSP70B promoter control.

### 3.5. Heat-Only-Activated, KRT1 Promoter-Restricted (Second-Generation) RCCVs Are Stringently Heat-Regulated and Are Broadly Protective Vaccines

**Figure 4:**
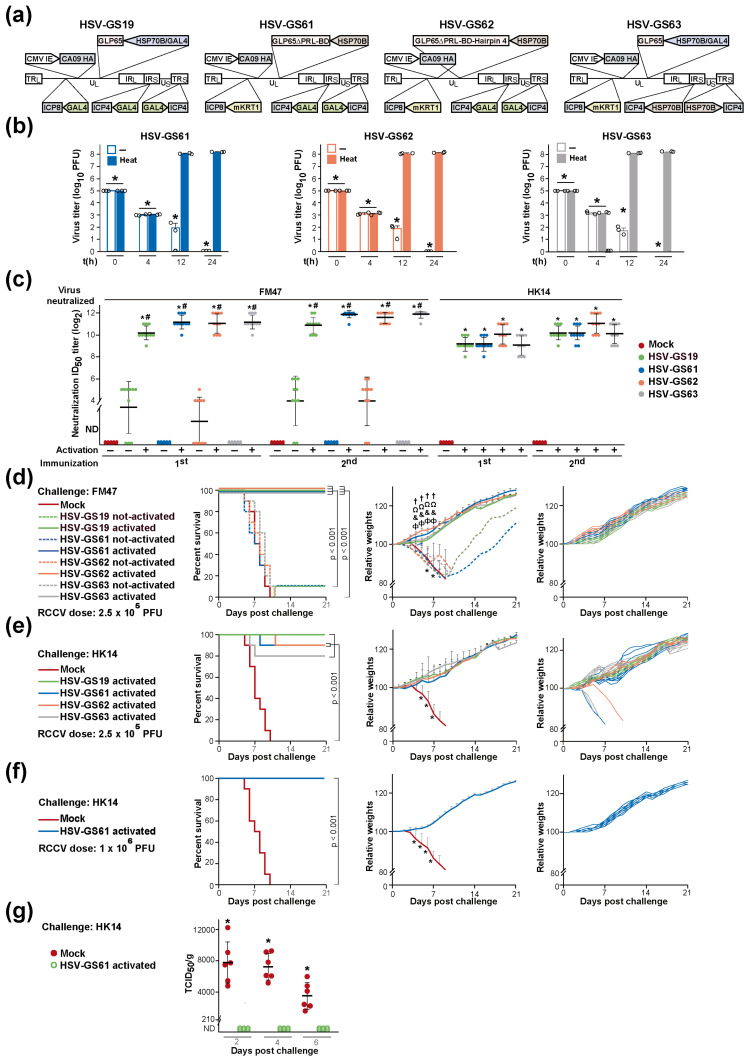
Heat-regulated replication of RCCVs HSV-GS61, HSV-GS62, and HSV-GS63, neutralizing antibody and protective responses induced by vaccination with the latter RCCVs, and lung viral titers after challenge. (**a**) Diagrammatic structures of RCCVs HSV-GS19, HSV-GS61, HSV-GS62, and HSV-GS63. See Figure 1 for additional explanations. (**b**) Single-step growth experiments with RCCVs HSV-GS61 (**left**), HSV-GS62 (**center**), and HSV-GS63 (**right**). For each RCCV, two sets of parallel confluent cultures of HEK293T cells were infected with the RCCV at an MOI of 3. One set was heat-treated by partially immersing the sealed dishes in a 43.5 °C water bath for 30 min (heat). All cultures were then incubated further at 37 °C. At 0, 4, 12, and 24 h post-treatment, three dishes from each set were removed, and virus was harvested from cells and medium. Infectious virus from each culture was titered on E5 cells transfected 24 h prior with ICP8 expression plasmid pICP8. Data are presented as total PFU. * *p* ≤ 0.05, comparing to the heat-activated group at 12 or 24 h. (**c**) Neutralizing antibodies. Groups (n = 10) of adult BALB/c mice were pre-bled and bled 3 wk after the first immunization with the indicated RCCV (each administered at 2.5 × 10^5^ PFU/animal) or vehicle (mock) and, again, 3 wk after the second immunization (immediately prior to challenge), and sera were prepared. Serial 2-fold dilutions of 1:16-diluted sera were analyzed for neutralizing antibodies against the indicated influenza virus strains by the microneutralization assay described in ref. [12]. Data are presented as neutralization ID_50_ titers (reciprocal dilutions where infection was reduced by 50% relative to normal serum expressed as geometric mean ID_50_). ND: not detected (below the limit of detection). *p* ≤ 0.05, comparing to the mock group (*) or the not-activated RCCV group (#). (**d**,**e**) Challenge experiments. Groups (n = 10) of adult BALB/c mice were inoculated on their rear footpads with 2.5 × 10^5^ PFU/animal of RCCVs HSV-GS19, HSV-GS61, HSV-GS62, or HSV-GS63, or vehicle (mock). As part of the activation treatment, HSV-GS19-inoculated animals received ulipristal (50 µg/kg) IP at the time of inoculation and, again, one day later. Three hours after inoculation, all animals of the indicated groups (i.e., the groups labeled as immunized with an activated RCCV) were subjected to a ten-minute heat treatment of their hindlimbs at 44.5 ˚C. This immunization and activation procedure was repeated 3 wk later. Three weeks after the second immunization, all animals were challenged intranasally with a lethal dose of either influenza virus strain FM47(H1N1) (**d**) or mouse-adapted strain HK14(H3N2) (**e**). Animals were observed daily, and weights were recorded. Left graphs: survival (≤20% weight loss) after challenge; center graphs: averaged relative weights of surviving animals after challenge. Weights are relative to weights on the day of the challenge. Relative values and standard deviations are shown; *p* ≤ 0.05, comparing activated and not-activated HSV-GS19 (†), HSV-GS61 (Φ), HSV-GS62 (Ω), or HSV-GS63 (&) groups or comparing the mock-treated group and either of the activated HSV-GS19, HSV-GS61, HSV-GS62, and HSV-GS63 groups (*); right graphs: relative weights after the challenge of all animals of the groups immunized with activated RCCVs. Weights are relative to weights on the day of the challenge. (**f**) Further challenge experiment performed as described in (d-e), except that the dose of RCCV HSV-GS61 administered was 1 × 10^6^ PFU/animal. Twice-HSV-GS61-immunized or mock-immunized animals were challenged with a lethal dose of influenza virus HK14(H3N2). * *p* ≤ 0.05, comparing to the HSV-GS61-immunized group. (**g**) Lung titers of challenge virus HK14. The results are presented as TCID_50_ values per g of tissue. Limit of detection: 210 TCID_50_/g tissue. ND: not detected. * *p* ≤ 0.05, comparing to groups immunized with RCCV.

RCCVs containing the HSP70B promoter-controlled gene for AP-binding site-deleted GLP65 (HSV-GS61) or containing the same HSP70B promoter-controlled TA gene comprising an inserted hairpin 4 sequence (HSV-GS62) were generated by replacement of the HSP70B/GAL4-GLP65 cassette in HSV-GS19/mKRT1 with the respective modified cassettes (Figure 4a). As an alternative approach to increasing the stringency of heat regulation, the GAL4 promoters of the ICP4 genes in HSV-GS19/mKRT1 were exchanged for HSP70B promoters, generating recombinant HSV-GS63. It is noted that HSV-GS63 retains the HSP70B/GAL4-GLP65 cassette of HSV-GS19/mKRT1 but no longer contains any TA-controlled gene.

Whether RCCVs HSV-GS61, HSV-GS62, and HSV-GS63 replicated in a strictly heat-regulated fashion was explored by single-step growth experiments in HEK293T cells (RCCV-infected at an MOI of 3). All RCCVs were found to replicate efficiently upon activation by transient heat treatment (at 43.5 °C for 30 min) (Figure 4b). The recombinants did not detectably replicate in the absence of activation. HSV-GS61, HSV-GS62, and HSV-GS63 were also tested for their ability to replicate in neurons using a line of differentiated human neurons (LUHMES). We were unable to detect any of the RCCVs in culture supernatants by plaque assay at 24 h post heat induction.

In an experiment to find out whether the replication of the heat-only-activated RCCVs was also stringently heat-controlled in vivo, two sets of groups of three adult BALB/c mice were inoculated on their rear footpads with 2.5 × 10^5^ PFU/animal of RCCV HSV-GS19 or one of RCCVs HSV-GS61, HSV-GS62, and HSV-GS63. Three hours after inoculation, all mice of one set were subjected to a ten-minute heat treatment of their hindlimbs at 44.5 °C. Ulipristal (50 µg/kg) was administered IP to HSV-GS19-inoculated mice that were to be heat-treated at the time of inoculation and again on the subsequent day. Animals of both sets were euthanized 48 h after virus inoculation, and DNA was extracted from their hindfeet and quantified by qPCR using a probe and primers specific for viral DNA polymerase. The results revealed that the replication of RCCVs HSV-GS61, 62, and 63 in the mouse feet had essentially only occurred subsequent to activating heat treatment (Table 2).

**Figure 5:**
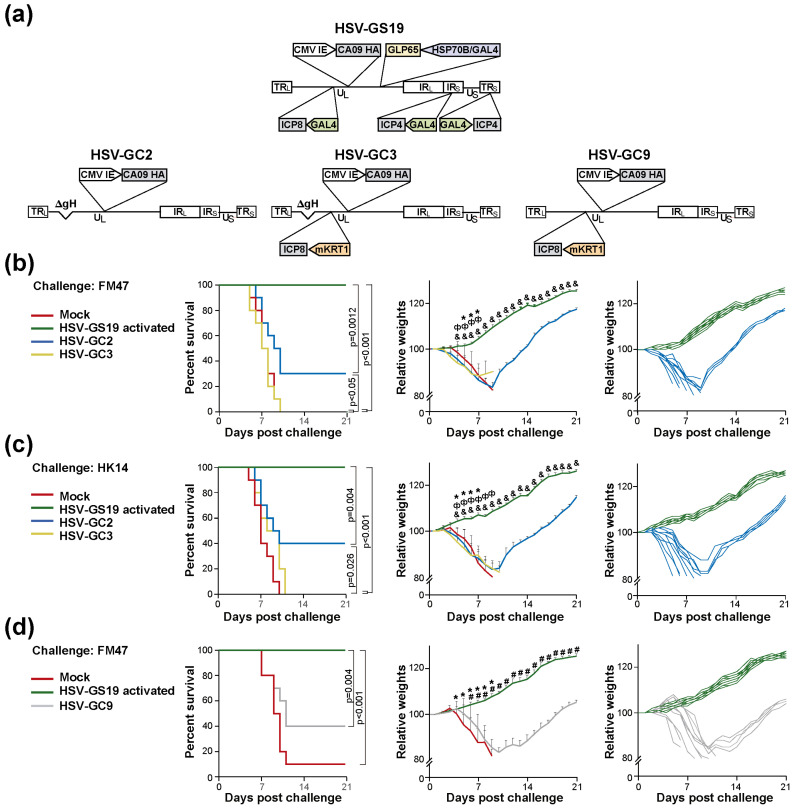
Challenge experiments to compare the vaccine efficacies of RCCV HSV-GS19 and recombinants HSV-GC2, HSV-GC3, and HSV-GC9. (**a**) Diagrammatic representation of the structures of HSV-GS19 and recombinants HSV-GC2, 3, and 9. Deletion of the gH gene is indicated (ΔgH). See Figure 1 for additional explanations. (**b**,**c**) Challenge experiment. Groups (n = 10) of adult BALB/c mice were immunized twice with 2.5 × 10^5^ PFU/animal of RCCV HSV-GS19 (heat- and AP-co-activated), recombinants HSV-GC2 or HSV-GC3, or vehicle (mock), as described in Figure 4d,e. Animals were challenged intranasally with influenza virus strain FM47(H1N1) in (**b**) or (HK14)(H3N2) in (**c**). Left graphs: survival (≤ 20% weight loss) after challenge; center graphs: averaged relative weights of surviving animals after challenge. Weights are relative to weights on the day of the challenge. Relative values and standard deviations are shown. *p* ≤ 0.05, comparing the activated HSV-GS19 group and the mock group (*) or the activated HSV-GS19 group and the HSV-GC2 (&) or the HSV-GC3 (Φ) group; right graphs: relative weights after the challenge of all animals of the RCCV- and HSV-GC-immunized groups. Weights are relative to weights on the day of the challenge. (**d**) Further challenge experiment. Animals twice-immunized with HSV-GC9 or activated HSV-GS19 or vehicle were challenged with influenza virus strain FM47(H1N1). See (**b**,**c**) for details. Note that in the center graph, relative weights for the mock group are only shown down to the nadir. *p* ≤ 0.05, comparing the activated HSV-GS19 group and the mock group (*) or the HSV-GC9 group (#).

Next, we compared the vaccine efficacies of the heat-only-activated RCCVs HSV-GS61, HSV-GS62, and HSV-GS63 with that of the heat- and AP-co-activated RCCV HSV-GS19. In the large experiment from which the results presented in Figure 4c–e and Figure 5b,c were obtained, sets of triplicate groups of adult BALB/c mice (n = 10/group) were inoculated epidermally on their rear footpads with 2.5 × 10^5^ PFU/animal of HSV-GS19, HSV-GS61, HSV-GS62, or HSV-GS63. Animals of two additional groups (n = 10) were inoculated with the vehicle (mock). As part of the activation treatment, the animals of two of the HSV-GS19 groups also received IP injections of 50 µg/kg of body weight of ulipristal at the time of virus inoculation and on the following day. Three hours after RCCV inoculation, all animals of two groups of each set (including the latter two HSV-GS19 groups) were subjected to a 44.5 °C/ten-minute heat treatment to their hindlimbs. Three weeks after the first immunization, all groups were immunized a second time. After a further 3 wk, all animals of the not-heat-treated group and one of the two heat-treated groups of each set were challenged intranasally with a lethal dose of heterologous group 1 influenza virus strain FM47(H1N1). As a reminder, all RCCVs expressed the HA of group 1 influenza virus strain CA09(H1N1). All animals of the other heat-treated group of each set were similarly challenged with mouse-adapted group 2 influenza virus strain HK14(H3N2). The mock-immunized groups were also challenged with the group 1 and the group 2 influenza virus strain. All animals were observed daily, and weights were recorded.

Serum samples were obtained from animals of each group pre-immunization, 3 wk after the first immunization and 3 wk after the second immunization (immediately prior to the challenges). Microneutralization assays revealed robust serum titers of antibodies neutralizing both group 1 influenza virus strain FM47(H1N1) and group 2 influenza virus strain HK14(H3N2) in animals immunized once or twice with heat-activated RCCVs HSV-GS61, HSV-GS62, or HSV-GS63 (Figure 4c). Titers were comparable with those in sera from animals immunized with activated HSV-GS19. Neutralizing antibodies were not detected in sera from mock-immunized animals, and neutralizing antibody titers were exceedingly low in sera from animals that had been immunized once or twice with not-activated recombinants. Neutralizing antibodies were not detected in sera from pre-bleeds.

Immunization with heat-activated RCCVs HSV-GS61, HSV-GS62, or HSV-GS63 protected animals fully against a lethal challenge with the heterologous group 1 strain FM47 (Figure 4d, left graph). HSV-GS61 and HSV-GS62 also afforded nearly complete protection (90%) against a lethal challenge with group 2 strain HK14, and HSV-GS63 was only insignificantly less efficacious (Figure 4e, left graph). As demonstrated for HSV-GS61, a modest increase in vaccine dose sufficed to generate a fully protective response (Figure 4f). Little or no protective effects were induced by immunization with not-activated RCCVs (Figure 4d, left graph). For the groups of animals immunized with activated RCCVs and challenged with group 1 strain FM47, no weight losses but steady weight gains were observed following the challenge (Figure 4d, center graph). At the level of individual animals of these groups, none of the animals immunized with activated RCCVs HSV-GS61, HSV-GS62, or HSV-GS63 lost weight but all gained weight during the 3 wk following the challenge (Figure 4d, right graph). The same observation was made for all RCCV-immunized animals that survived the challenge with influenza virus strain HK14 (Figure 4e,f, center and right graphs). We concluded that the heat-only-activated, tissue-restricted RCCVs are effective and broadly protective influenza vaccines. Their efficacy is only insignificantly lower than that of heat- and AP-co-activated RCCVs. The apparent slight deficit in efficacy can be compensated by increasing the vaccine dose.

Viral titers in the lungs of challenged animals were assessed in a further experiment. One group of adult BALB/c mice (n = 30) was immunized twice with activated recombinant HSV-GS61 (1.0 × 10^6^ PFU/animal), and another group was mock-immunized. At 2, 4, and 6 days after intranasal challenge with a lethal dose of influenza virus strain HK14, six animals of each of the groups were euthanized, lungs were recovered, and virus extracted from lung tissues was titered on MDCK cells. Whereas elevated titers of the challenge virus were determined in mock-immunized animals at all time points (Figure 4g), the results revealed that essentially no replication of the virus occurred in the lungs of the vaccinated animals. Ten animals from each group were observed daily for 3 wk, and survival was recorded. All animals of the vaccinated group but none of the mock-immunized group survived the challenge.

### 3.6. Substitution or Elimination of the Heat Control Is Associated with a Substantial Loss of Vaccine Efficacy

We further explored whether the heat-activated gene switch controlling the replication of RCCVs HSV-GS61-63 may be replaced by a self-limiting mechanism, eliminating the requirement of heat treatment as part of the vaccination procedure. The heat-activated gene switch serves to confine RCCV replication to the inoculation region as well as to limit the replication to a single cycle. HSVs deleted for certain envelope genes (e.g., the gH gene), so-called disabled infectious single cycle (DISC) viruses, are capable of undergoing a single round of replication to produce non-infectious progeny virus [20]. Recombinant HSV-GC2 was derived from wild-type HSV-1 strain 17syn+ by deletion of the gH gene and insertion of the same expressible HA gene that is present in recombinant HSV-GS19 and HSV-GS61-63 (Figure 5a). To further contain replication within the skin administration region, the native promoter of the ICP8 gene of recombinant HSV-GC2 was replaced with an mKRT1 promoter. The resulting recombinant was named HSV-GC3. The performance of the latter recombinants in a lethal challenge experiment is documented in Figure 5b,c. Immunization (twice) with recombinant HSV-GC2 protected only a minority of animals against a lethal challenge by influenza virus group 1 strain FM47 or by group 2 strain HK14 (left graphs). Recombinant HSV-GC3 did not protect at all against the lethal challenges. A low level of protection by HSV-GC3 was observed in a second experiment that employed somewhat lower doses of challenge viruses. All surviving HSV-GC2-immunized animals experienced weight loss (center and right graphs). Vaccination with HSV-GC2 or HSV-GC3 did not appear to induce antibodies capable of neutralizing the challenge viruses.

Finally, we asked whether the low vaccine efficacy of recombinants HSV-GC2 and HSV-GC3 was a consequence of the deletion of the gH gene. We generated recombinant HSV-GC9 by inserting the same expressible HA gene that is present in the other recombinants into the genome of recombinant 17syn+/mKRT1. Recombinant HSV-GC9 is functionally equivalent to RCCVs HSV-GS61-63 except that its replication in permissive cells is constitutive rather than heat-regulated. Immunization (twice) with recombinant HSV-GC9 only protected a minority of animals against a lethal challenge by influenza virus strain FM47 (Figure 5d, left graph). All surviving animals suffered weight loss (center and right graphs). These results suggested that the heat switch control of replication (or heat treatment) cannot be substituted or eliminated without greatly reducing vaccine efficacy.

## 4. Discussion

We previously described RCCV-based vaccines that protect more broadly against influenza viruses than currently used vaccines as well as any experimental HA-directed vaccine described to date [12,21,22,23,24,25,26,27,28,29,30,31,32,33,34,35,36,37,38,39,40,41,42]. The replication of these RCCVs was controlled by a two-tiered mechanism provided by a gene switch that is co-activated by heat and an AP. The heat control was intended to limit as well as confine virus replication to the inoculation region, guarding against uncontrolled local proliferation as well as dissemination and systemic replication. The AP co-control was aimed at averting replication in latently infected nerve cells as well as systemic replication under conditions of stress. Although HSV-1 is associated with relatively low morbidity and mortality, both systemic infection and infection of the CNS are potential dangers, in particular for immunodeficient individuals. Controlling replication at two levels is considered to contribute importantly to vaccine safety. While overlapping, the two aspects of the control mechanism do not contribute equally. The heat control alone may be incapable of safely suppressing replication in latently infected nerve cells or rendering systemic replication impossible in an individual experiencing stress, e.g., a severe fever. The drug co-control is insufficiently localized to restrict replication to the inoculation site, even if the drug is administered locally. It is noted that owing to the nature of the mechanism that controls their replication, RCCV vaccines should also be inherently safe for administration to immunocompromised subjects.

Protocols for conventional vaccination are necessarily less complex than protocols that would need to be implemented for an RCCV-based vaccination, the latter involving the administration of an AP at the time of RCCV inoculation and the application of a heat treatment shortly after inoculation. Even if the inconvenience of having to administer a drug could be avoided by combining the vaccine and drug in a single formulation, the added cost of the drug may be a significant obstacle to the introduction of the vaccine approach. Perhaps, more importantly, the administration to certain populations, e.g., the very young, of a drug for a purpose that is only indirectly related to therapy may be considered undesirable or unacceptable. The administration of an AP to women of reproductive age may also be contraindicated. Hence, it was of considerable, perhaps critical, importance to attempt to replace the drug co-control with a mechanism that achieves the same goal but does not involve a drug.

We opted to replace the heat- and AP-activated gene switch with a heat-only-activated gene switch for the control of the replication-essential ICP4 genes and to employ a highly tissue-selective promoter (a KRT1 promoter) expected not to be active in the nervous system and most other tissues but highly active in keratinocytes for controlling the replication-essential ICP8 gene. A promoter that is selectively active in keratinocytes was chosen because epidermal administration was considered a key aspect of RCCV-based vaccination. Efficient but limited replication of RCCVs is critical to their efficacity as vaccines, as evidenced by experiments described herein as well as in our previous publications [11,12]. Keratinocytes in the skin and in mucosal membranes are the natural host cells of lytic HSVs (from one of which RCCVs are derived), and the viruses replicate efficiently in these cells. An epidermal route of administration also renders the heat activation of RCCVs readily achievable. Heat can be directed to regions in the skin without a need for complex technology. In contrast, the delivery of an appropriate heat dose to a more deep-seated tissue is far more demanding. For example, technologies such as high-intensity focused ultrasound in combination with MRI, PET, or ultrasound imaging have been used for the controlled heating of interior body regions (discussed in ref. [43]). The skin as a barrier region is an immunologically active organ [44]. Epidermal administration by skin scarification is a classical mode of vaccine delivery and has been the preferred procedure used for smallpox vaccination. A recent comparative study revealed that application to the skin by scarification is a particularly advantageous route of administration of vaccines against respiratory disease, generating more abundant T cells, including lung-resident memory T cells, than other routes [45]. It is noted that epidermal administration may be achieved by means of microneedle patches, avoiding the pain associated with skin scarification as well as open lesions and the associated danger of infection and contact transmission to immunocompromised individuals. Microneedle patches delivering live measles virus, an enveloped virus, have been developed [46]. The technology employed may be adapted for producing microneedle patches comprising live RCCVs.

We found that KRT1 promoters are essentially inactive in human and mouse neuron-derived cell lines and other non-keratinocyte cell lines. It is noted that mouse as well as human KRT1 promoters were essentially inactive in both mouse and human neuroblastoma cells. We recognized that no matter how many cell lines we were to test, we could never have been quite certain that an RCCV co-controlled by a KRT1 promoter was functionally replication-deficient in the nervous system. We, therefore, addressed the question using a highly sensitive in vivo assay of neurovirulence, the mouse footpad lethal challenge model. A recombinant (17syn+/mKRT1) that was identical to the highly virulent HSV-1 strain 17syn+ except that its ICP8 gene was controlled by a KRT1 promoter was found not to cause any short-term neurotoxicity even at doses that exceeded the LD_50_ of 17syn+ by several hundred-fold. The same recombinant replicated well in mouse feet to which it had been administered but could not be detected in brain tissues. In other experiments, mice were immunized twice with recombinant HSV-GC9, a derivative of 17syn+/mKRT1 further containing an expressible gene for the HA of influenza virus strain CA09(H1N1). The vaccine dose applied corresponded to several hundred times the LD_50_ of strain 17syn+. No neurotoxicity or distress was noted 6 wk after administration. We concluded that the KRT1 promoter control effectively prevented functionally relevant replication in the nervous system.

There may have been other ways of preventing the replication of an RCCV in the nervous system although not necessarily of confining its replication to the epidermis. Perhaps the most obvious approach would have been to disable the thymidine kinase (TK) gene in an RCCV. The deletion of this gene has long been known to sharply reduce the neurovirulence of HSVs [47,48]. However, a more recent study has demonstrated that TK-deficient HSV-1 strains are capable of persistently infecting neural tissues of nude mice as well as replicating in these tissues [49]. Another viral neurovirulence gene, e.g., infected cell protein 0 (ICP0), may have been disabled. A virus lacking the ICP0 function may not be capable of establishing latency in nerve cells [50]. Another possibility may have been to introduce mutations in the genes for glycoprotein K and membrane protein UL20, mutations which have been reported to prevent virus entry into distal axons of ganglionic neurons and may, thereby, preclude significant reactivation [51]. We have no information on whether the disablement of the ICP0 gene or of the glycoprotein K and UL20 genes will prevent replication in neural cells of immunocompromised subjects. Yet another possibility that could be explored would be to introduce in an RCCV a gene expressing, under the control of a neural cell-selective promoter [52], an antisense transcript that could block the expression of a critical viral or host cell gene. Instead of an antisense transcript, a ribozyme may be expressed. An LAT (latency-associated transcript)-targeting ribozyme that reduces reactivation from latency has been described previously [53]. Another approach could be to introduce into an RCCV a gene expressing a repressor of a viral or host cell gene under the control of a neural-selective promoter. A repressor of viral early genes may be constructed by fusing sequences from ICP4 (including those encoding the protein’s DNA-binding domain) and a Kruppel-associated box (KRAB) repressor domain.

We also thought to replace the heat control of the RCCVs with a mechanism that does not involve heat treatment. DISC viruses resemble (once) heat-activated RCCVs in that they only undergo a single round of replication. They differ from heat-activated RCCVs in that their replication is not restricted to the administration site and their progeny is incapable of newly infecting cells. Challenge experiments were conducted with recombinant HSV-GC2, a DISC virus derived from the wild-type HSV-1 strain 17syn+ by deletion of the gH gene and introduction of the same expressible HA gene that is present in the RCCVs. The recombinant was found to be only poorly effective as an influenza vaccine. Recombinant HSV-GC3 that further contains an mKRT1 promoter-controlled ICP8 had an even lower efficacity. These results suggested that the heat treatment that triggers the efficient transient replication of the RCCVs may play an additional role that contributes to vaccine efficacy, perhaps enhancing HA folding and/or presentation or stimulating relevant aspects of the immune system [54]. This notion was further supported by the observation that recombinant HSV-GC9 is also a poor vaccine. The main difference between vaccination with recombinant HSV-GC9 and vaccination with RCCVs HSV-GS61-63 is the heat treatment that is applied to activate the replication of the RCCVs. It is noted that activated RCCVs replicate with similar but not higher efficiency than the wild-type HSV-1 strain 17syn+ [10]. We conclude that the heat control of RCCV-based influenza vaccines may not be readily substituted. Besides their low efficacy, there would be safety concerns with vaccines such as HSV-GC9. Conceivably, they may cause persistent infections in the skin and, perhaps, even in the mouth and genital tissues (and other non-neural tissues in which the KRT1 promoter may have residual activity), especially in immunocompromised subjects. Furthermore, they would rely on a single control mechanism for safety.

As mentioned above, the heat activation of an RCCV in a skin site may be readily accomplished. We previously reported proof-of-principle experiments employing a simple technology that is inexpensive and is capable of safely delivering to the skin of a human subject a heat dose that will be adequate for activating a heat-controlled RCCV [43]. The technology envisages administering an RCCV-based vaccine to an extremity of a human subject. A short time later, a small heating pad is fastened to the extremity to cover the inoculation region. Upon activation, the device will deliver an appropriate heat dose to the skin in the inoculation region. The heating pad essentially consists of a small polyvinyl chloride (PVC) bag containing a stabilized supercooled solution of sodium thiosulfate pentahydrate, a readily available, inexpensive, and nontoxic salt with a melting point of around 48 °C. When triggered mechanically, the salt solution in the heating pad begins to crystallize, delivering heat at a constant temperature of about 45 °C to its surface for 15 min. It is noted that the heating pad can be stored at room temperature and operates independently of an energy supply. If desired, a spent heating pad can be regenerated by immersing it in 60–80 °C water until the salt has completely melted.

There may be concerns about the efficacy of RCCV-based vaccines in subjects previously infected with an HSV. Numerous previous studies addressing this issue concluded that preexisting immunity to HSV should not be an obstacle to the use of HSV vectors as vaccines or oncolytic agents [55,56,57,58,59,60,61,62]. More relevant to influenza vaccines, a recent study demonstrated that vaccination with an (non-replicating) HSV vector expressing an influenza virus HA elicited a protective immune response against the homologous influenza virus regardless of preexisting immunity against HSV [63].

## 5. Conclusions

We have elaborated second-generation RCCV-based influenza vaccines that are controlled at two levels and that can be heat-activated to undergo efficient but limited replication in a skin administration site. In a mouse model, the activated vaccines that express a group 1 HA induce robust titers of broadly neutralizing antibodies and afford protection against heterologous and cross-group influenza virus challenges. These results as well as those obtained with the original heat- and drug-co-activated RCCVs [12] may provide a basis for advanced investigations aimed at developing an RCCV-based universal flu vaccine. As was also noted before [12], the robustness of RCCV-based influenza vaccines may be enhanced by the expression of multiple different HAs.

## Figures and Tables

**Table 1:** RCCVs and non-RCCV recombinants employed in the present study.

RCCV	Non-RCCV Recombinant	Description
HSV-GS19		ICP4 and ICP8 genes controlled by a heat-activated and antiprogestin-armed gene switch
HSV-GS19/mKRT1		ICP4 genes controlled by a heat-activated and antiprogestin-armed gene switch; ICP8 gene under the control of a keratin 1 gene promoter
	17syn+/mKRT1	ICP8 gene under the control of a keratin 1 gene promoter
	HSV-GC9	ICP8 gene under the control of a keratin 1 gene promoter
HSV-GS61		ICP4 genes controlled by a heat-induced, unregulated transactivator; ICP8 gene under the control of a keratin 1 gene promoter
HSV-GS62		ICP4 genes controlled by a heat-induced, unregulated transactivator (expressed at a lower level than by HSV-GS61); ICP8 gene under the control of a keratin 1 gene promoter
HSV-GS63		ICP4 genes controlled by heat shock promoters; ICP8 gene under the control of a keratin 1 gene promoter
	HSV-GC2	Gene for glycoprotein H deleted
	HSV-GC3	Gene for glycoprotein H deleted; ICP8 gene under the control of a keratin 1 gene promoter

All recombinants were derived from wild-type HSV-1 strain 17syn+. All recombinants except 17syn+/mKRT1 express the HA of influenza virus strain A/California/07/2009 (CA09).

**Table 2:** Heat-regulated replication of RCCVs HSV-GS61, HSV-GS62, and HSV-GS63 in mouse feet.

Vaccine	HSV-GS19	HSV-GS61	* HSV-GS62	HSV-GS63
Not-activated	ND	ND	44 ± 8	ND
Activated	2.3 ± 0.4 × 10^3^	1.3 ± 0.9 × 10^3^	2.1 ± 0.3 × 10^4^	4.9 ± 1.0 × 10^3^

Results are presented as relative genomes/mg tissue and standard deviations. ND: not detected. Sensitivity: 0.15 relative genomes/mg tissue. See the text for a description of the experiment and the Materials and Methods for experimental detail. * Determined in a separate assay.

## Data Availability

Data presented in this study are available on reasonable request from the corresponding author.
